# Tuberculosis-Associated Death among Adult Wild Boars, Spain, 2009–2014

**DOI:** 10.3201/eid2212.160677

**Published:** 2016-12

**Authors:** Jose A. Barasona, Pelayo Acevedo, Iratxe Diez-Delgado, Joao Queiros, Ricardo Carrasco-García, Christian Gortazar, Joaquín Vicente

**Affiliations:** Instituto de Investigación en Recursos Cinegéticos, Ciudad Real, Spain (J.A. Barasona, P. Acevedo, I. Diez-Delgado, J. Queiros, R. Carrasco-Garcia, C. Gortazar, J. Vicente);; Universidad Complutense de Madrid, Madrid, Spain (J.A. Barasona, I. Diez-Delgado);; Centro de Investigacão em Biodiversidade e Recursos Genéticos, Vairão, Portugal (J. Queiros);; Faculdade de Ciências da Universidade do Porto, Porto, Portugal (J. Queiros)

**Keywords:** Disease control, disease-related mortality, GPS monitoring, Mycobacterium tuberculosis complex, wild boar, wildlife surveillance, tuberculosis and other mycobacteria, bacteria, geographic information systems, zoonoses, TB

## Abstract

We investigated adult Eurasian wild boar (*Sus scrofa*) survival and death in 2 tuberculosis-endemic populations with different harvest pressure in Spain. Overall, tuberculosis accounted for 30% of total deaths. Increased survival in protected areas has direct implications for wild boar management and tuberculosis control.

Eurasian wild boar (*Sus scrofa*) population dynamics and hunting strategies might influence the persistence of disease (*1*). Determining the death rates for wild boar and unfolding the relative contribution of several causes of death and their nature (additive vs. compensatory death) is key to predicting the effects of harvesting, predation, and disease on population dynamics over time and to develop disease control–oriented hunting strategies. In central and northern Europe, the effect of disease-mediated death on wild boars is relatively low, whereas predation, winter starvation, and especially hunting play more important roles (*2**,**3*). In Mediterranean regions, natural death from summer starvation during droughts has been described, but most deaths are attributed to hunting (*4*); no information is available about rates of disease-related death among wild boars.

Animal tuberculosis (TB) caused by the *Mycobacterium tuberculosis* complex (MTC) is a reemerging multihost infectious disease (*5*). In Spain, the Eurasian wild boar is regarded as the key wildlife MTC maintenance host; its infection prevalence rates are >50% in Mediterranean areas that have dense wild boar populations (*6*). Up to one third of wild boar piglets might become infected during their first 6 months of life (*7*). In half of MTC-infected wild boars, generalized lesions develop that affect the lungs, particularly in juveniles (12–24 months of age). In adults (>2 years), the observed proportion of wild boars with generalized TB decreases, suggesting some degree of TB-driven death among juveniles (*6**,**8*). TB is a sporadic cause of death among wild boars (*9*), but no data are available about its actual contribution to mortality.

In the context of growing and expanding wild boar populations and of increasing concern about the effect of wild boar infections (*10*), we hypothesized that TB could be a major component of total wild boar death in Spain and have implications for TB control and wildlife management. We aimed to 1) describe the rates and causes of adult wild boar death and 2) compare the total death and its causes in 2 TB-endemic regions that differ in harvest pressure.

## The Study

We compared 2 settings: a mosaic of game estates and a protected area. Montes de Toledo (MT) is a mountain chain in the central Spanish plateau whose large game estates are mainly devoted to recreational hunting. Harvest is conducted by dog-driven hunts; average annual extraction quota is 2.26 wild boar/km^2^ and no age or sex are selected (i.e., extraction is random). Doñana National Park (DNP) is a protected area on the Atlantic coast of southern Spain. Harvest is part of population control management because no recreational hunting is allowed within the park; this modality has a minimal extraction capacity (1.11 wild boar/km^2^) and targets wild boar seen by park rangers (random and opportunistic). Wild boars, except piglets, have no natural predators (occasionally stray dogs) in the study areas.

During 2009–2014, we captured (*11*) and fitted very high frequency global positioning system–global system for mobile communications (VHF-GPS-GSM) collars (Microsensory, Spain) to 45 free-ranging adult wild boars (24 from MT and 21 from DNP; Technical Appendix Table) following Animal Experimentation legislation (PR-2015-03-08). We collected serum and tested it for antibodies to MTC by using ELISA (89.6% sensitivity [*12*]). Post-release monitoring was programmed to acquire 1 GPS location per hour. We monitored the animals daily for death (alarm set at 12 h of inactivity) to promptly retrieve carcasses and assess the cause of death. The 18 retrieved carcasses underwent a full postmortem examination, and tissue samples (pooled lymph nodes and lung) were submitted for culture (Technical Appendix).

We detected serum antibodies to MTC in 35 (78%) of 45 GPS-collared wild boars. We found no differences in MTC serum antibody prevalence between study sites (p>0.05 by Fisher exact test) and no differences in survival time between antibody-positive and -negative wild boars (p>0.05 by Mann-Whitney U test). MTC infection was confirmed by culture in 13 (72%) of 18 wild boars for which postmortem results were available. The 9 wild boars that died of generalized TB had severe lesions in >1 anatomic region; >70% of lung tissue was affected (Technical Appendix Figure 1).

We assessed total survival probability and the main causes of death (Figure). The mean annual death rate (45.48% ± 5.6% SE overall) was higher in the regularly hunted MT (56%) than in the protected DNP (34%). Overall, harvest accounted for half (53%) of total annual deaths, whereas TB contributed to 30% of deaths. The remaining 17% of deaths were caused by predation (stray dogs in DNP) and unknown causes (Table). The mean annual death rate for adult wild boars caused by harvest was significantly higher in MT (40%) than in the protected DNP (8%; Fisher exact test, p = 0.011). However, death from TB did not differ between MT (12%) and DNP (14%; p>0.05 by Fisher exact test) (Technical Appendix Table).

**Figure:**
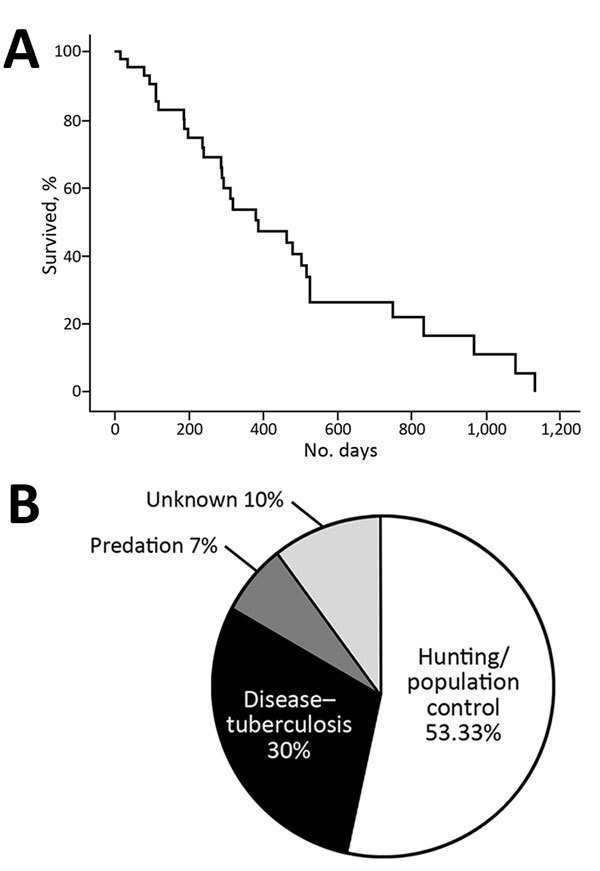
Total survival probability and the main causes of death among wild boars (*Sus scrofa*), Spain, 2009–2014. A) Kaplan-Meier survival curve representing the proportion of adult wild boars alive over time for all the animals studied. B) Percentage of each cause of death among wild boars (i.e., when considering only all dead animals).

**Table:** Causes of death of GPS-collared adult wild boars (*Sus scrofa*), Spain, 2009–2014*

Variable	Study site		Total
	Montes de Toledo†	Doñana National Park‡	
No. GPS-collared wild boars	24	21	45
Mean survival time ± SE, d§	297.28 ± 40.91	672.78 ± 96.53	470.43 ± 58.69
Mean annual survival rate ± SE, %¶	44.17 ± 7.55	66.35 ± 7.73	54.52 ± 5.60
Mean annual mortality rate ± SE, %¶	55.83 ± 7.55	33.64 ± 7.73	45.48 ± 5.60
Proportion of deaths, %			
From harvest	72.22	25	53.33
From tuberculosis	22.22	41.67	30.00
From other causes	5.56	33.93	16.67

*GPS, global positioning system. †High harvest pressure.  ‡Low harvest pressure.  §Mantel-Cox log-rank, χ^2^ = 11.42, 1 d.f., p = 0.001. ¶Mann-Whitney U test, U = −1.994, p = 0.046.

Mean survival time was twice as long in DNP (average 672 ± 96 days) as in MT (297 ± 41 days; Mantel-Cox, χ^2^ = 11.42, 1 d.f.; p = 0.001). Kaplan-Meier survival probabilities and causes of death are detailed by study area in Technical Appendix Figure 2. Two death peaks were found, 1 in summer (July) associated with TB and 1 in autumn (October–January) associated with harvest (Technical Appendix Figure 3).

## Conclusions

The results confirmed our hypothesis that TB causes a substantial proportion of deaths among adult wild boars in TB-endemic Mediterranean areas of Spain. This information is relevant for TB control at the wildlife–livestock interface and for understanding wild boar and TB dynamics under different harvest pressure.

Severely diseased wild boars, with advanced generalized TB lesions affecting large proportions of the lung, probably are important shedders of MTC (super-shedders [*13*]). The higher survival rate for MTC super-shedders in protected areas, such as DNP, resulting from a low harvest pressure might contribute toward explaining the extremely high spread of TB in sites where risky artificial management, such as feeding, is absent (*5*).

The large proportion of natural death from TB (30% of deaths) contrasts with results obtained in other parts of Europe (total natural death rate 3% [*3*]), although this finding is consistent with differences in TB prevalence within Europe (*14*). Previous findings suggest that TB-induced death is relevant in subadults but decreases in adults (*6**,**8*), hence, deaths of juvenile wild boars deserves special study. However, given the chronic nature of TB and the early reproduction of wild boars, TB is unlikely to substantially contribute to wild boar population regulation. In fact, we observed a mean annual death rate of 45%, which is below the recommended annual harvest or death rate of 65% needed to maintain stable wild boar populations (*3**,**15*).

Two additional aspects about hunting and wild boar TB deserve attention. First, increased hunting might contribute to TB control in wild boars by reducing population size and by reducing survival of super-shedders. Hunting bans should therefore be reconsidered in protected areas in which TB is a concern. Second, TB causes a substantial loss of adult (trophy) wild boars, thus reducing the profitability of the hunting industry. Hunters should therefore actively engage in TB control.

Technical Appendix Statistical analyses; list of global positioning system (GPS)–collared adult wild boars; details of GPS-collared wild boar found dead; survival curves and causes of death; and monthly death distribution of GPS-collared wild boars.
