# Supplementary material for: Tuberculosis-Associated Death among Adult Wild Boars, Spain, 2009–2014
Source: Emerg Infect Dis. 2016 Dec;22(12):2178–80. doi: 10.3201/eid2212.160677 (PMC5189151; doi:10.3201/eid2212.160677)
Supplement: Technical Appendix — Statistical analyses; list of global positioning system (GPS)–collared adult wild boars; details of GPS-collared wild boar found dead; survival curves and causes of death; and monthly death distribution of GPS-collared wild boars. [file 16-0677-Techapp-s1.pdf]

Article DOI: http://dx.doi.org/10.3201/eid2212.160677

Tuberculosis-Associated Death among Adult Wild Boars, Spain, 2009–2014

Technical Appendix

Statistical Analyses

Death rates were explored by estimating the daily probability of survival (*P*) according to method proposed by Kenward (*1*). Annual death rates were calculated as $1 - P^{365}$, which is the probability that an individual boar will die in any given year. In addition, Kaplan-Meier survival curves were used to compute the probabilities of death occurring, given those animals that had died, along with those animals with "censored" observations (i.e., still alive or nontransmitting collars) at the conclusion of the study (*2*). We calculated the annual death rates and Kaplan-Meier survival curves for the entire population, in addition to those for Montes de Toledo and Doñana National Park independently. We used the Mantel-Cox log-rank, Mann-Whitney U, and Fisher exact test analyses at a level of significance of 0.05 to test survival differences between areas, sex, and according to the tuberculosis test at the time of the first capture. Data were analyzed by using IBM SPSS statistical package version 20 (IBM Corporation, Somar, NY, USA).

References

1. Kenward RE. A manual for wildlife radio tagging. San Diego: Academic Press; 2001.

2. Nuss K, Warneke M. Life span, reproductive output, and reproductive opportunity in captive Goeldi's monkeys (*Callimico goeldii*). Zoo Biol. 2010;29:1–15. PubMed

Technical Appendix Table. List of GPS-collared adult wild boars (*Sus scrofa*), Spain, 2009–2014

Animal ID	Study area	Sex	Cause of death or monitoring discontinued	No. days monitored	*Mycobacterium tuberculosis* ELISA
1	MT	F	Disease–tuberculosis	33	Positive
2	MT	F	Disease–tuberculosis	109	Positive
3	MT	F	Hunting	502	Positive
4	MT	F	Hunting	186	Positive
5	MT	F	Hunting	462	Positive
6	MT	F	Hunting	197	Positive
7	MT	F	Hunting	379	Negative
10	MT	F	Unknown	515	Negative
8	MT	F	Monitoring discontinued	16	Positive
9	MT	F	Monitoring discontinued	181	Positive
11	MT	M	Disease–tuberculosis	110	Positive
12	MT	M	Disease–tuberculosis	288	Positive
13	MT	M	Hunting	78	Positive
14	MT	M	Hunting	235	Positive
15	MT	M	Hunting	525	Negative
16	MT	M	Hunting	478	Positive
17	MT	M	Hunting	311	Negative
18	MT	M	Hunting	526	Negative
19	MT	M	Hunting	14	Negative
20	MT	M	Hunting	184	Positive
21	MT	M	Monitoring discontinued	55	Positive
22	MT	M	Monitoring discontinued	165	Positive
23	MT	M	Monitoring discontinued	38	Negative
24	MT	M	Monitoring discontinued	16	Negative
25	DNP	F	Disease–tuberculosis	285	Negative
26	DNP	F	Disease–tuberculosis	238	Positive
27	DNP	F	Disease–tuberculosis	749	Positive
28	DNP	F	Population control	92	Positive
30	DNP	F	Unknown	117	Positive
31	DNP	F	Unknown	386	Positive
29	DNP	F	Monitoring discontinued	54	Positive
32	DNP	F	Monitoring discontinued	739	Positive
33	DNP	M	Disease–tuberculosis	292	Positive
34	DNP	M	Disease–tuberculosis	317	Positive
35	DNP	M	Population control	831	Positive
36	DNP	M	Population control	967	Positive
37	DNP	M	Predation	1131	Positive
38	DNP	M	Predation	1079	Positive
39	DNP	M	Monitoring discontinued	304	Positive
40	DNP	M	Monitoring discontinued	220	Positive
41	DNP	M	Monitoring discontinued	176	Positive
42	DNP	M	Monitoring discontinued	523	Positive
43	DNP	M	Monitoring discontinued	759	Negative
44	DNP	M	Monitoring discontinued	268	Positive
45	DNP	M	Monitoring discontinued	425	Positive

DNP, Doñana National Park; GPS, global positioning system; ID, identification; MT, Montes de Toledo.

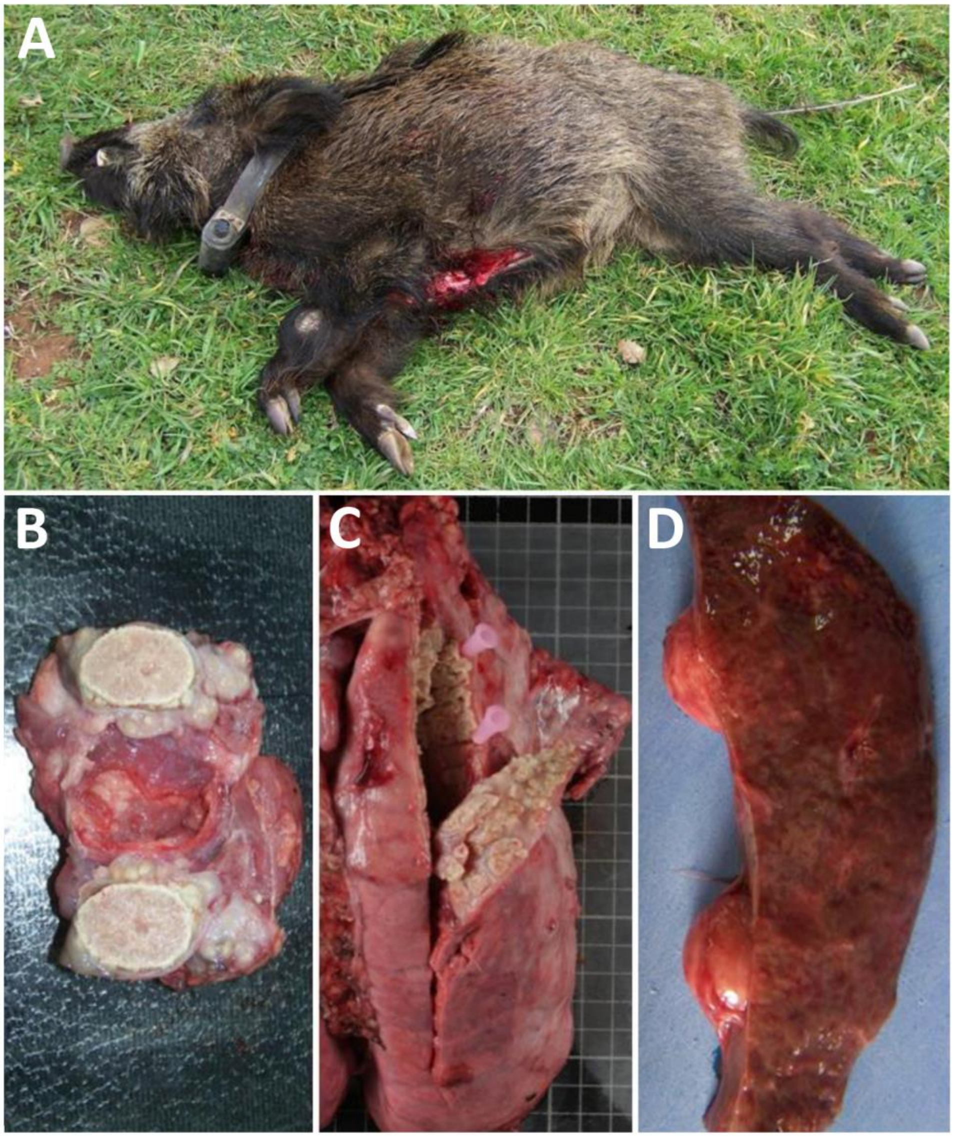

Technical Appendix Figure 1. A) Global positioning system–collared wild boar (*Sus scrofa*) found dead as the result of generalized tuberculosis on a cattle farm in the Montes de Toledo, Spain. B) Calcified and caseous lesions in the mandibular lymph node (B), lung (C), and spleen (D) of this boar.

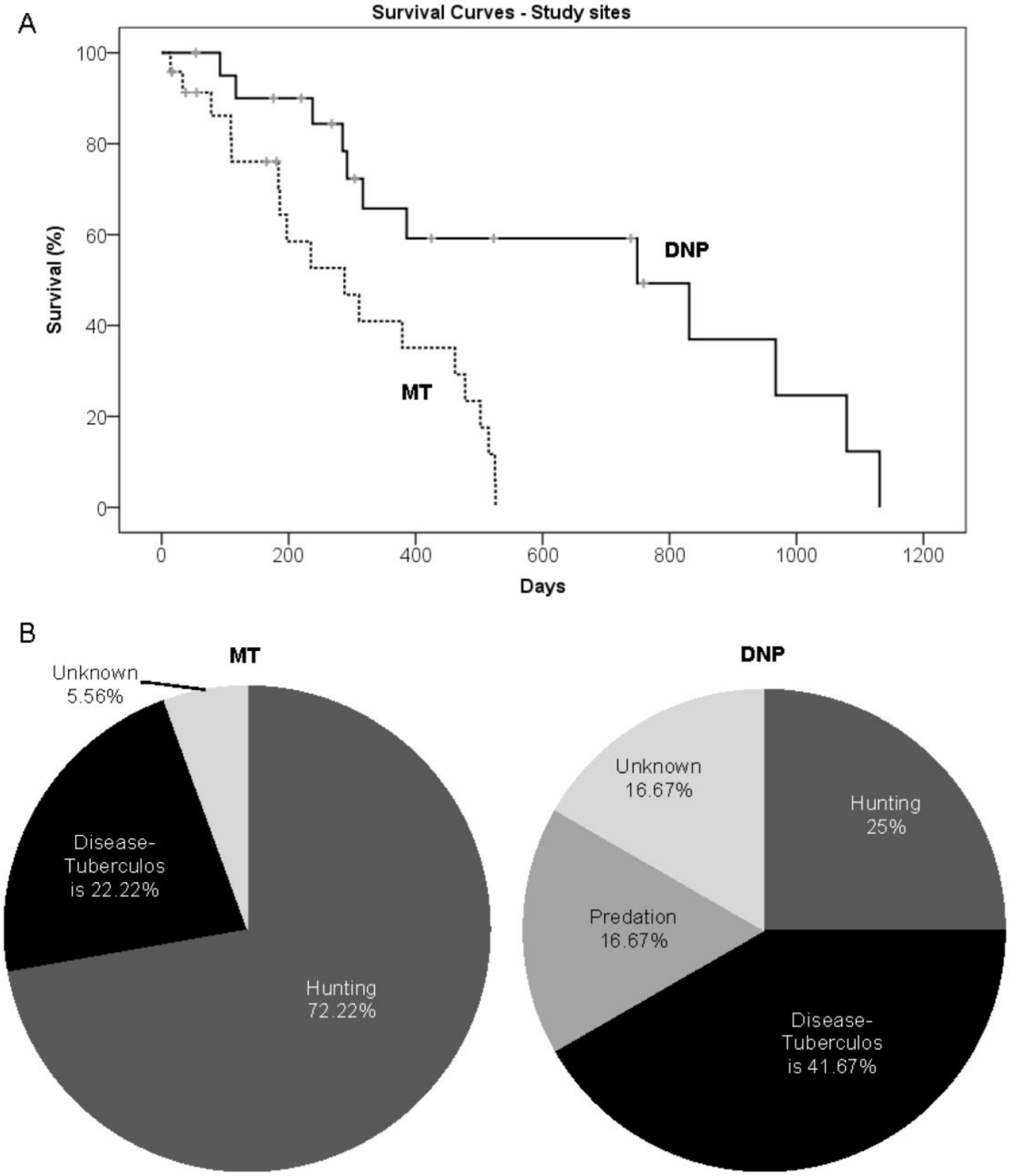

Technical Appendix Figure 2. A) Kaplan-Meier survival curves representing the proportion of free-ranging wild boars (*Sus scrofa*) alive over time for all the animals studied in each area. Differences in probability of survival between Montes de Toledo (MT) and Doñana National Park (DNP) are significant (Mantel-Cox χi^2 = 11.42, 1 d.f., p = 0.001). Tick marks on each curve indicate a specific censored animal, with some ticks overlapping each other. B) Percentage of each cause of death among wild boars (i.e., when considering only all dead animals) in MT and DNP.

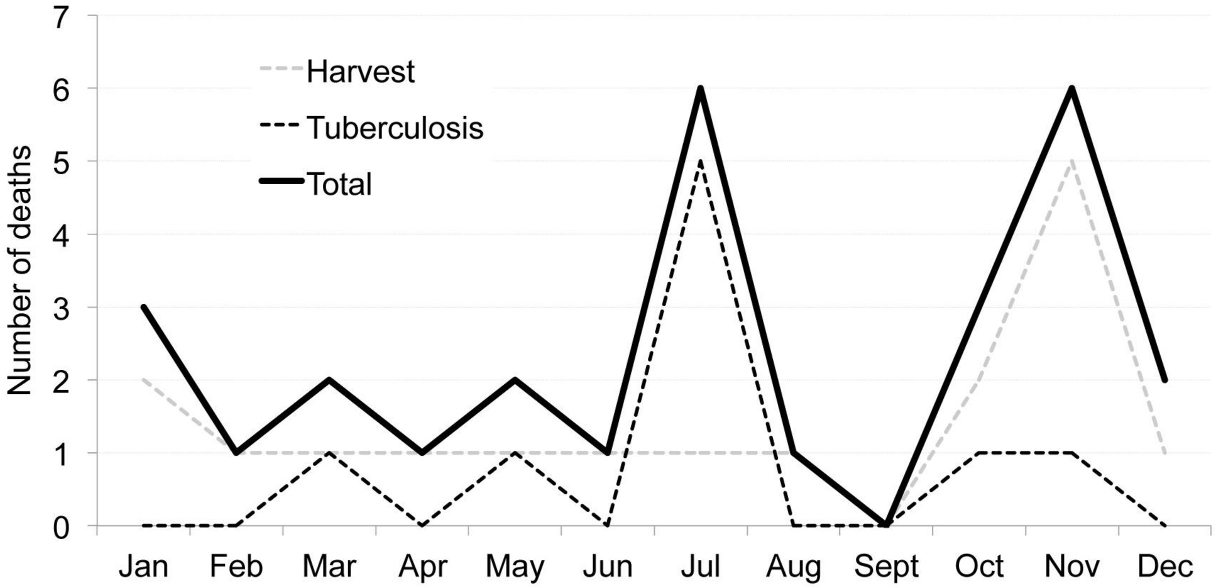

Technical Appendix Figure 3. Monthly death distribution of global positioning system–collared adult wild boars (*Sus scrofa*) by main known causes, Spain, 2009–2014.
